# *UBE2C* expression is elevated in hepatoblastoma and correlates with inferior patient survival

**DOI:** 10.3389/fgene.2023.1170940

**Published:** 2023-06-12

**Authors:** Ruth Nousiainen, Katja Eloranta, Noora Isoaho, Stefano Cairo, David B. Wilson, Markku Heikinheimo, Marjut Pihlajoki

**Affiliations:** ^1^ Pediatric Research Center, Children’s Hospital, Helsinki University Hospital, University of Helsinki, Helsinki, Finland; ^2^Division of Micro and Nanosystems, School of Electrical Engineering and Computer Science, KTH Royal Institute of Technology, Stockholm, Sweden; ^3^ Champions Oncology, Hackensack, NJ, United States; ^4^ Istituto di Ricerca Pediatrica, Padova, Italy; ^5^ XenTech, Evry, France; ^6^Department of Developmental Biology, Washington University School of Medicine, St. Louis, MO, United States; ^7^Department of Pediatrics, Washington University School of Medicine, St. Louis Children’s Hospital, St. Louis, MO, United States; ^8^Faculty of Medicine and Health Technology, Center for Child, Adolescent and Maternal Health Research, Tampere University, Tampere, Finland

**Keywords:** hepatoblastoma, liver tumor, pediatric cancer, ubiquitin, *UBE2C*

## Abstract

Hepatoblastoma (HB) is the most common malignant liver tumor among children. To gain insight into the pathobiology of HB, we performed RNA sequence analysis on 5 patient-derived xenograft lines (HB-243, HB-279, HB-282, HB-284, HB-295) and 1 immortalized cell line (HUH6). Using cultured hepatocytes as a control, we found 2,868 genes that were differentially expressed in all of the HB lines on mRNA level. The most upregulated genes were *ODAM*, *TRIM71*, and *IGDCC3*, and the most downregulated were *SAA1*, *SAA2*, and *NNMT*. Protein-protein interaction analysis identified ubiquitination as a key pathway dysregulated in HB. *UBE2C*, encoding an E2 ubiquitin ligase often overexpressed in cancer cells, was markedly upregulated in 5 of the 6 HB cell lines. Validation studies confirmed UBE2C immunostaining in 20 of 25 HB tumor specimens *versus* 1 of 6 normal liver samples. The silencing of *UBE2C* in two HB cell models resulted in decreased cell viability. RNA sequencing analysis showed alterations in cell cycle regulation after *UBE2C* knockdown. *UBE2C* expression in HB correlated with inferior patient survival. We conclude that *UBE2C* may hold prognostic utility in HB and that the ubiquitin pathway is a potential therapeutic target in this tumor.

## 1 Introduction

Hepatoblastoma (HB) is the most common malignant pediatric liver neoplasm with an annual incidence of 1.9/1,000,000 (Aronson and Meyers, 2016; Feng et al., 2019) that has been increasing over the past decades (Linabery and Ross, 2008). The etiology of most cases of HB remains unknown, but preterm birth, birthweight less than 2500 g, and certain genetic conditions such as Familial Adenomatous Polyposis and Beckwith-Wiedemann syndrome are associated with increased risk of HB (Spector and Birch, 2012; Paquette et al., 2019). Wnt/β-catenin signaling has been identified as one of the pathways altered in the majority of HB tumors, but other molecular pathways involved in HB pathogenesis are not yet fully understood (Udatsu et al., 2001; Armengol et al., 2011). Current treatment of HB includes complete surgical resection combined with doxorubicin and cisplatin- or carboplatin-based neoadjuvant and adjuvant chemotherapy (Zsíros et al., 2010). These forms of chemotherapy are effective but often lead to serious long-term side effects including cardiotoxicity, ototoxicity, and nephrotoxicity (Spector and Birch, 2012; Volkova and Russell, 2012). Although the prognosis of HB has improved over the years, 20%–30% of HB patients still respond poorly to current treatment modalities (Sivaprakasam et al., 2011), so new therapeutic targets are needed.

Metabolic reprogramming is one of the hallmarks of cancer (Faubert et al., 2020). Highly proliferating tumor cells need to adapt to conditions such as hypoxia and lack of nutrients and thus require metabolic reprogramming to enhance their survival. Many of the oncogenes and tumor suppressors are participating in dysregulation of metabolic pathways in cancer (Nong et al., 2023). Also, genes coding for metabolic enzymes have been described to be mutated or aberrantly expressed in several tumor types (Sreedhar and Zhao, 2018). Major changes include alterations in glucose metabolism, known as Warburg effect, as well as in amino acid and lipid metabolism (Counihan et al., 2018). Alterations in genes regulating ubiquitination and deubiquitination and their role as modulators of the metabolic changes of tumor cells are also known to be essential in cancer progression (Sun et al., 2020).

Some changes in metabolic genes have already been demonstrated to be present in HB. In HB tumors, activating mutations in the Wnt/β-catenin pathway genes lead to altered glucose metabolism mediated by upregulation of *GLUT3* (Crippa et al., 2017). Immortalized HepG2 cells, originally derived from a HB, exhibit deranged bile acid metabolism (Kullak-Ublick et al., 1996) due in part to and downregulated *SLC10A1* (Wang et al., 2020). In the same study, the authors also found that downregulation of *SLC10A1* resulted in upregulated adenosine metabolism. Retinol metabolism and cytochrome P450 pathway have both been demonstrated to be downregulated in HB (Sekiguchi et al., 2020). Despite these findings, the gene expression behind the metabolic alterations taking place in HB still remain poorly understood.

The objective of this study was to characterize the landscape of metabolic genes in HB using RNA sequencing data and bioinformatics analyses. Our overarching goal was to identify potential treatment targets and novel biomarkers in HB.

## 2 Materials and methods

### 2.1 RNA sequencing and microarray datasets

Raw RNA sequencing datasets from previously published studies were obtained from Gene Expression Omnibus (GEO) database of National Center for Biotechnology Information (NCBI) (http://www.ncbi.nlm.nih.gov/geo/) and European Genome-phenome Archive (EGA) (https://ega-archive.org/). Accession numbers were as follows: EGAS00001004827/EGAD00001006621 (HB-282, HB-295, HB-279, HB-284, HB-243), GSE140520 (PHH-D3, 1–3), GSE83518 (1HUH6HB, 2HUH6HB). Raw microarray data of gene expression in 53 HB tissue samples and 14 noncancerous liver tissue samples collected from the HB patients at the time of surgery were acquired from GEO, accession number GSE131329.

### 2.2 Identification of differentially expressed genes and differentially expressed metabolic genes

RNA sequencing dataset files were analyzed with Chipster software (https://chipster.rahtiapp.fi/) (Kallio et al., 2011). Reads were preprocessed using Trimmomatic and aligned to human reference genome (GRCh38) using HISAT2 (Kim et al., 2015). Reads per genes were counted with HTseq (Anders et al., 2015). Differential expression analysis was conducted with the edgeR package (Robinson et al., 2009). Differentially expressed genes (DEGs) were then filtered using cut-off criteria adjusted to *p*-value <0.05 and |log_2_FC|≥1.0.

Microarray data were analyzed with Chipster plus the normalization tool for Affymetrix gene arrays (Li, 2001; Irizarry et al., 2003). Statistical tests were conducted using the “Two group tests” tool (empirical Bayes as test and BH as *p*-value adjustment method) (Smyth, 2004).

Human metabolic genes were obtained from The Virtual Metabolic Human database (VMH) (Supplementary Table S1) (Noronha et al., 2019). A list of DEGs in each cell line was compared to a list of human metabolic genes to further filter the results. Duplicates were removed from the list leaving 3,285 unique genes.

### 2.3 Protein-protein interaction (PPI) network construction

PPI networks of differentially expressed metabolic genes in each cell line were constructed with the online tool of STRING database (Szklarczyk et al., 2019) using 0.7 as minimum required interaction score. Results were visualized with Cytoscape (Shannon et al., 2003). Highly interconnected areas (clusters) of these interaction maps were identified and scored using the Cytoscape plugin Molecular Complex Detection (MCODE) (Bader and Hogue, 2003) using the following criteria: degree cut-off: 2; haircut: yes; fluff: no; node score cut-off: 0.2; K-core: 2; max. Depth: 100. Based on this score, highest-ranking cluster of each cell line was chosen to be investigated more thoroughly. PPI network of UBE2C was constructed using STRING with 0.4 as minimum required interaction score.

### 2.4 Kyoto encyclopedia of genes and genomes (KEGG) and gene ontology (GO) pathway enrichment analyses

Genes in each cell line’s highest-scoring cluster were uploaded to Enrichr (Chen et al., 2013; Kuleshov et al., 2016; Xie et al., 2021). Results of KEGG pathway and GO Biological Processes term enrichment analyses were imported from the website. Results with adjusted *p*-value of <0.05 were considered significant. The top 5 terms with lowest adjusted *p*-values from both KEGG and GO were chosen for each HB cell line. Python programming language (Python Software Foundation) with Matplotlib (Hunter, 2007), NumPy (Harris et al., 2020), and pandas (McKinney, 2010) libraries were used for handling and plotting this data.

### 2.5 Statistical analysis of clinical variables

Student’s t-test, Mann-Whitney U test, and receiver operating characteristic (ROC) curves were used to analyze microarray gene expression data and clinical variables provided in GSE131329 dataset. Statistical significance was set to *p*-value <0.05. Analyses were conducted with R software (v. 4.0.3) or GraphPad Prism (v. 8.4.2; GraphPad, San Diego, CA, United States).

### 2.6 Gene co-expression analysis

An online tool for gene co-expression analysis, GeneFriends v 5.0 (Raina et al., 2022), was used to analyze the gene co-expression of *UBE2C*. Following input parameters were used: Species; *Homo Sapiens*, Data Source; SRA, Tissue: All tissue types, Object type; Gene, Seed gene: *UBE2C*, Pearson correlation threshold: 0.75.

### 2.7 Patient samples

Formalin-fixed paraffin-embedded (FFPE) HB tumor samples [*n* = 24, median age in years 3.37 (range 0.23–11.75)] and normal liver control samples (NL, *n* = 6, median age in years 12.5 (range 3–26)) were obtained from the Helsinki Biobank at Helsinki University Hospital. HB samples were originally collected at the time of surgical treatment from patients treated in Children’s Hospital, Helsinki University Hospital between 1 January 1991, and 31 December 2017. Prior to resection, majority of patients had received preoperative chemotherapy. NL samples were collected from liver transplantation donors in Helsinki University Hospital. This study was approved by Helsinki University Hospital institutional ethical committee (HUS/3319/2018) and conducted in accordance with Finnish bylaws. Informed consent was obtained when samples were deposited to the Helsinki Biobank.

### 2.8 Immunohistochemistry

Samples were heated for 30 min at 60°C oven and deparaffinized with NeoClear (Merck-Millipore, Darmstadt, Germany). Target retrieval was performed using pH 9.0 target retrieval solution for 30 min at + 98°C (Dako, Glostrup, Denmark). Novolink Polymer Detection System Kit (Leica, Newcastle, United Kingdom) was used to block endogenous peroxidase activity and nonspecific binding. Samples were incubated with UBE2C polyclonal antibody at +4°C overnight (dilution 1:1,500; #PA5-102791; Invitrogen, Thermo Fisher Scientific). A polymerized reporter enzyme staining system (Novolink Polymer Detection System Kit) was used to visualize the bound antibody. UBE2C immunoreactivity was scored as strong nuclear staining (positive) or negative by two separate observers. Images were generated using 3DHISTECH Pannoramic 250 FLASH II digital slide scanner at Genome Biology Unit supported by HiLIFE and the Faculty of Medicine, University of Helsinki, and Biocenter Finland.

### 2.9 HB *in vitro* models

HB cell line HUH6 was obtained from Japanese Collection of Research Bioresources Cell Bank (Osaka, Japan). Cells were maintained in Dulbecco’s modified Eagle’s medium (DMEM)-glutaMAX (Gibco) supplemented with 10% FBS (Gibco), 100 U/mL penicillin (Gibco), and 100 μg/mL streptomycin sulfate (Gibco, Waltham, MA, United States). HB cell line HB-243 from patient-derived xenograft (PDX) was provided by XenTech (Evry, France) (Kats et al., 2019). HB-243 cells were cultured in Advanced DMEM/F12 (Gibco, Waltham, MA, United States) supplemented with 8% fetal bovine serum (FBS) (Gibco), 2 mM glutaMAX (Gibco), 100 U/mL penicillin (Gibco), 100 μg/mL streptomycin sulfate (Gibco) and 20 μM rock kinase inhibitor Y-27632 (S1049; SelleckChem, Houston, TX, United States). Absence of *mycoplasma* was regularly confirmed with PCR-based method (PromoCell, Heidelberg, Germany).

### 2.10 *UBE2C* silencing

*UBE2C* expression in HUH6 and HB-243 cell models was silenced by small interfering RNA (siRNA) transfection. The cells were exposed to 25 nM *UBE2C* ON-TARGETplus SMARTpool siRNA (cat# L-004693-00-0005) or ON-TARGETplus non-targeting (NT) control siRNA (cat# D-001810-10-05; both purchased from Horizon Discovery, Cambridge, United Kingdom). Dharmafect 4 (Horizon Discovery) was used to deliver the siRNAs into the HUH6 cells using the protocol provided. Knockdown efficacy was evaluated at mRNA and at protein level 48 h after transfection.

### 2.11 RNA and protein extraction

Total RNA and protein were extracted from cultured HUH6 and HB-243 cell models using NucleoSpin RNA/Protein extraction kit (Macherey-Nagel, Düren, Germany). Instructions provided by the manufacturer were followed.

### 2.12 Quantitative real-time polymerase chain reaction

Reverse transcription was carried out using iScript cDNA Synthesis Kit (Bio-Rad, Hercules, CA, United States). Quantitative polymerase chain reaction (qPCR) was performed using PowerUp SYBR Green Master Mix (Thermo Fisher Scientific, Fremont, CA, United States). The geometric mean of *B2M* and *PPIG* served as a reference. Primer sequenced were designed as follows: *B2M* 5′- GAT GAG TAT GCC TGC CGT GT—3′ (forward), 5′- CTG CTT ACA TGT CTT GAT CCC A- 3′ (reverse); *PPIG* 5′ -CAA TGG CCA ACA GAG GGA AG—3′(forward), 5′—CCA AAA ACA TGA TGC CCA—3′ (reverse); *UBE2C* 5′- CCG CCC GTA AAG G—3′ (forward), 5′- CTC AGG TCT TCA TAT ACT GTT CCA G -3′ (reverse).

### 2.13 Western blotting

Equal amounts of protein (10 µg) were loaded into Mini-Protein TGX stain-free gels (Bio-Rad) and separated using gel electrophoresis. Proteins were transferred to polyvinyl-fluoride membrane and 5% skimmed milk in Tris-buffered saline-Tween^20^ was utilized to block unspecific binding. Primary antibody incubation was performed at room temperature for overnight (UBE2C at dilution 1:500; #14234S; Cell Signaling Technology Inc., Danvers, MA, United States). Secondary antibody incubation was carried out at room temperature for 1 h (goat anti-rabbit IgG at dilution 1:10,000; #111-035-144, Jackson ImmunoResearch, West Grove, PA, United States). Protein bands were visualized using Enhanced Chemiluminescence detection kit (Amersham ECL reagent; GE Healthcare, Barrington, IL, United States). Protein quantification was performed with Image Lab software (version 6.0, Bio-Rad) by normalizing UBE2C band intensities to amount of total protein in corresponding lane utilizing stain-free technology (Gürtler et al., 2013).

### 2.14 RNA sequencing of *UBE2C* silenced HUH6 cells

HUH6 cells were cultured and treated with either *UBE2C* or non-targeting siRNAs. RNA and protein extraction were conducted as described above. Prior to sequencing, RNA concentration, quality, and integrity were assessed using Qubit fluorometer (Thermo Fisher Scientific, Waltham, MA, United States) and the TapeStation system (Agilent, Glostrup, Denmark). After quality assessment, RNA libraries were constructed applying polyA selection, and Illumina compatible cDNA libraries were prepared by GENEWIZ (Leipzig, Germany). Samples were then sequenced on Illumina NovaSeq 6,000 yielding 2 × 150 bp paired end reads (GENEWIZ). Processing of RNA sequencing data was done using Chipster software as described above. The cut-off criteria were set to adjusted *p*-value <0.1. No cut-off criteria were used for logFC. Enrichr was used to identify enriched pathways and ontologies.

### 2.15 Cell viability assays

Cell viability after *UBE2C* knockdown was evaluated with cell proliferation agent WST-1 and clonogenic survival assay. The WST-1 assay (Roche Diagnostics GmbH, Basel, Switzerland) was performed according to the manufacturer’s instructions at timepoint of 48 h. For the clonogenic assay, HUH6 cells transfected with *UBE2C* or NT siRNAs were disaggregated into single-cell suspension and seeded at low density into 12-well plates. After culturing for 72 h, the cells were washed with phosphate-buffered saline (PBS), fixed with 4% paraformaldehyde, permeabilized with 100% methanol, stained with crystal violet and rinsed with dH2O. The area occupied by cell colonies in each well was calculated using Cell Profiler (McQuin et al., 2018).

### 2.16 Migration assay

Cell migration was assessed using transwell migration inserts (8 um pore size; Merck Millipore, Darmstadt, Germany). The bottom of each insert was pre-coated with collagen I (0.1 mg/mL; Sigma Aldrich, St. Louis, MO, United States) and placed into 24-well plates containing cell culture medium (10% serum). *UBE2C* or NT siRNA transfected cells were seeded to upper side of membrane in starvation medium (serum-free) (seeding density 5 × 10^5^ cells per well). After culturing for 42 h, cells were fixed with 4% paraformaldehyde, permeabilized with 100% methanol, and stained with crystal violet. Non-migrated cells were removed from upper side of membrane with a cotton swab. In each insert, images were captured from five randomly chosen fields with Eclipse TS100 microscope supplemented with DS-Fi1 digital imaging system (magnification ×10; Nikon, Tokyo, Japan). The number of migrated cells was calculated with ImageJ software.

## 3 Results

### 3.1 Genes differentially expressed in HB cell lines vs. primary hepatocytes

**FIGURE 1:**
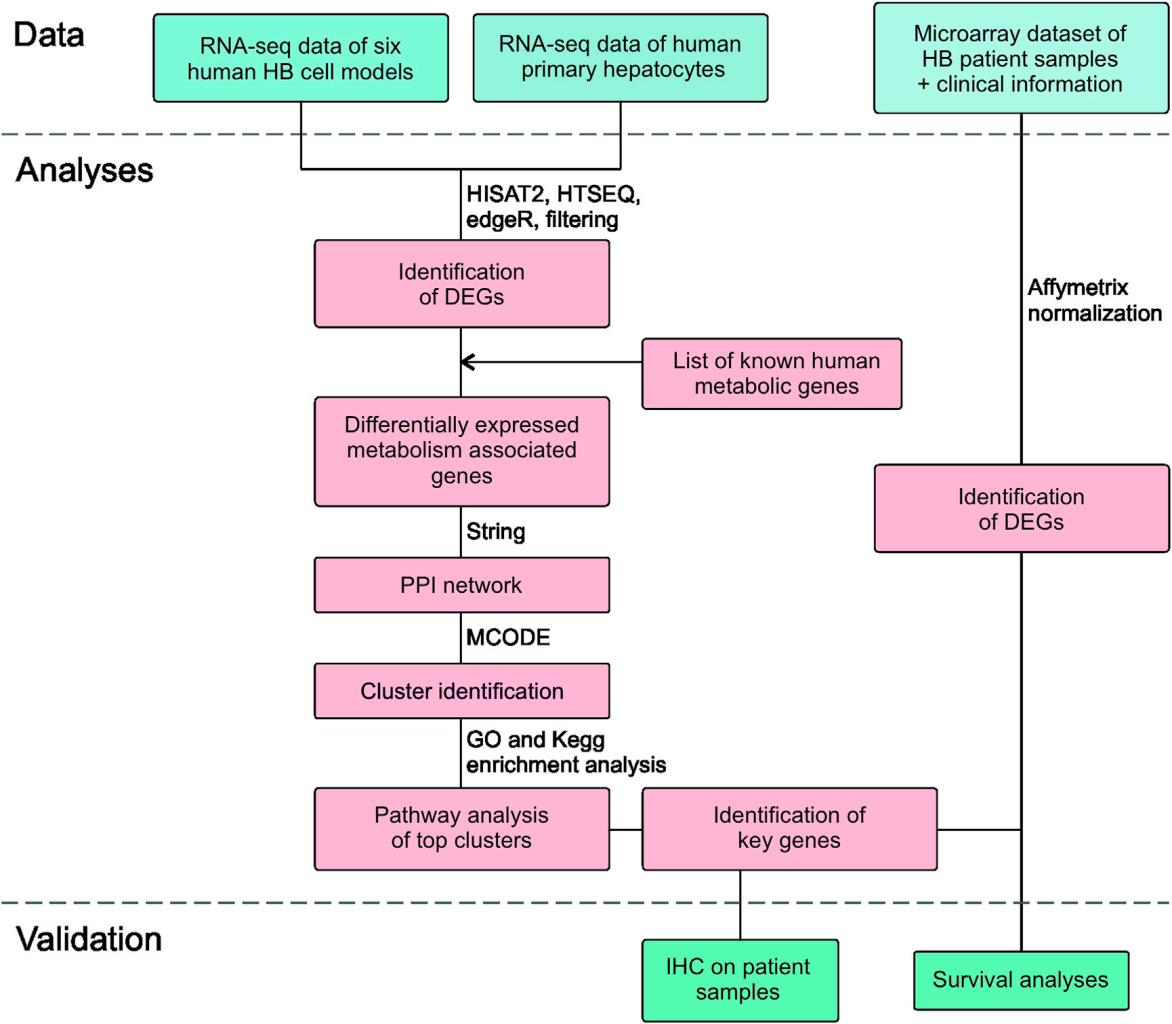
Flowchart of the study design.

**FIGURE 2:**
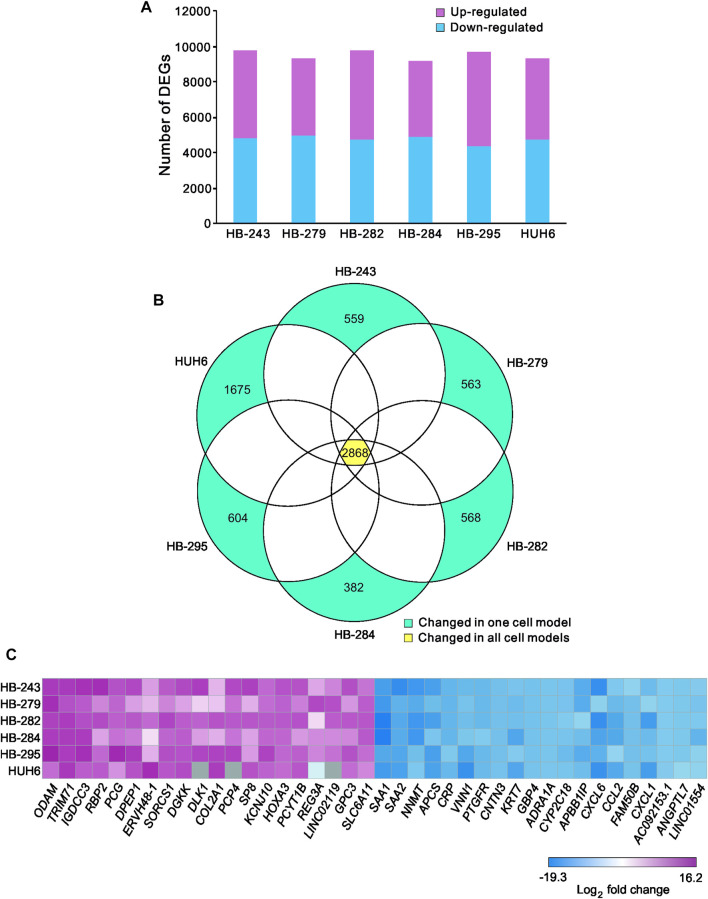
Differentially expressed genes in HB cell lines compared to primary hepatocytes Upregulated (pink) and downregulated (blue) genes in each HB cell line **(A)**. Venn diagram showing the number of significant differentially expressed genes compared to primary hepatocytes that were shared among all six HB cell models **(B)**. Heatmap of the 20 most downregulated and the 20 most upregulated differentially expressed genes sorted by log_2_FC **(C)**. Genes in gray = change not statistically significant. Differential expression analysis was conducted using the edgeR package.

The workflow is outlined in Figure 1. Five of the cell lines used in this study were established from aggressive HB tumors; the sixth was the immortalized human HB cell line, HUH6. Details of these cell lines are shown in Table 1 (Doi, 1976; Kats et al., 2019). RNA-sequencing analysis of these six cell lines identified approximately 9,000 differentially expressed genes (DEGs) in each cell line compared to primary hepatocytes (Figure 2A; Supplementary Table S2). Of these, approximately half were upregulated and half downregulated. Venn analysis showed that 2,868 of DEGs were shared among all 6 HB cell lines (Figure 2B). The top 20 most upregulated and most downregulated genes are shown in Figure 2C. The most upregulated genes were *ODAM, TRIM71,* and *IGDCC3*, while the most downregulated genes were *SAA1, SAA2,* and *NNMT*. Among the most upregulated genes were *GPC3*, *DLK1*, and *SP8*, previously connected to aggressive HB, underscoring the robustness of the analysis pipeline (Cairo et al., 2008; Zynger et al., 2008; Wagner et al., 2020).

**TABLE 1:** HB cell line characteristics.

Cell line	Age at sampling	Sex	Histology	Origin	References
HB-243	52 months	Male	embryonal	intrahepatic relapse	Kats et al., 2019
HB-279	79 months	Male	embryonal and macrotrabecular	primary tumor	Kats et al., 2019
HB-282	12 months	Male	embryonal	primary tumor	Kats et al., 2019
HB-284	83 months	Male	embryonal	peritoneal metastasis	Kats et al., 2019
HB-295	26 months	female	fetal	primary tumor	Kats et al., 2019
HUH6	12 months	Male	mixed, predominantly embryonal	primary tumor	Doi, (1976)

### 3.2 Differentially expressed metabolic genes

**FIGURE 3:**
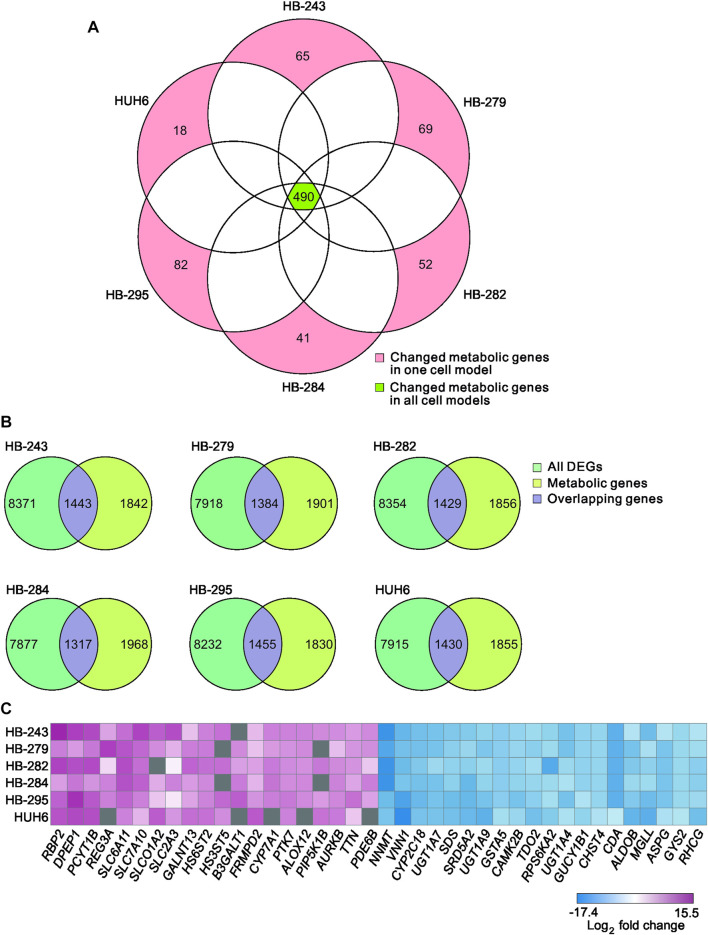
Differentially expressed metabolic genes in HB cell models Venn diagram showing the number of shared metabolic genes among the 6 HB cell lines **(A)**. Overlap of differentially expressed genes in each cell line and the list of all metabolic genes **(B)**. Heatmap of the 20 most up- and the 20 most downregulated metabolic genes in HB cell models **(C)**. Genes in gray = change not statistically significant.

A list of known human metabolic genes was obtained from Virtual Human Metabolomics (Supplementary Table S1). Venn analysis showed that 490 of these metabolic genes were shared among all 6 HB cell lines (Figure 3A; Supplementary Table S4). Approximately 1,400 DEGs in each HB cell line were classified as metabolic genes (Figure 3B; Supplementary Table S3). The 20 most upregulated and most downregulated DEGs overlapping with metabolic genes are listed in Figure 3C. The most upregulated metabolism-associated genes were *RBP2, DPEP1,* and *PCYT1B*, and the most downregulated genes were *NNMT, VNN1,* and *CYP2C18*. Out of these six genes, DPEP1, responsible for hydrolysis of several dipeptides, and nicotinamide N-methyltransferase NNMT have been demonstrated to play a role in HB pathogenesis in previous studies (Cui et al., 2019; Rivas et al., 2020). Other genes were not previously reported in HB. These genes are involved in regulating oxidative phosphorylation (*RBP2*, *VNN1*), phosphatidylcholine biosynthesis (*PCYT1B*), and xenobiotic and retinoid metabolism (*CYP2C18, RBP2*) (Chen and Goldstein, 2012; Grinde et al., 2014; Váraljai et al., 2015; Giessner et al., 2018; Blaner et al., 2020).

### 3.3 PPI-network construction and clustering

**FIGURE 4:**
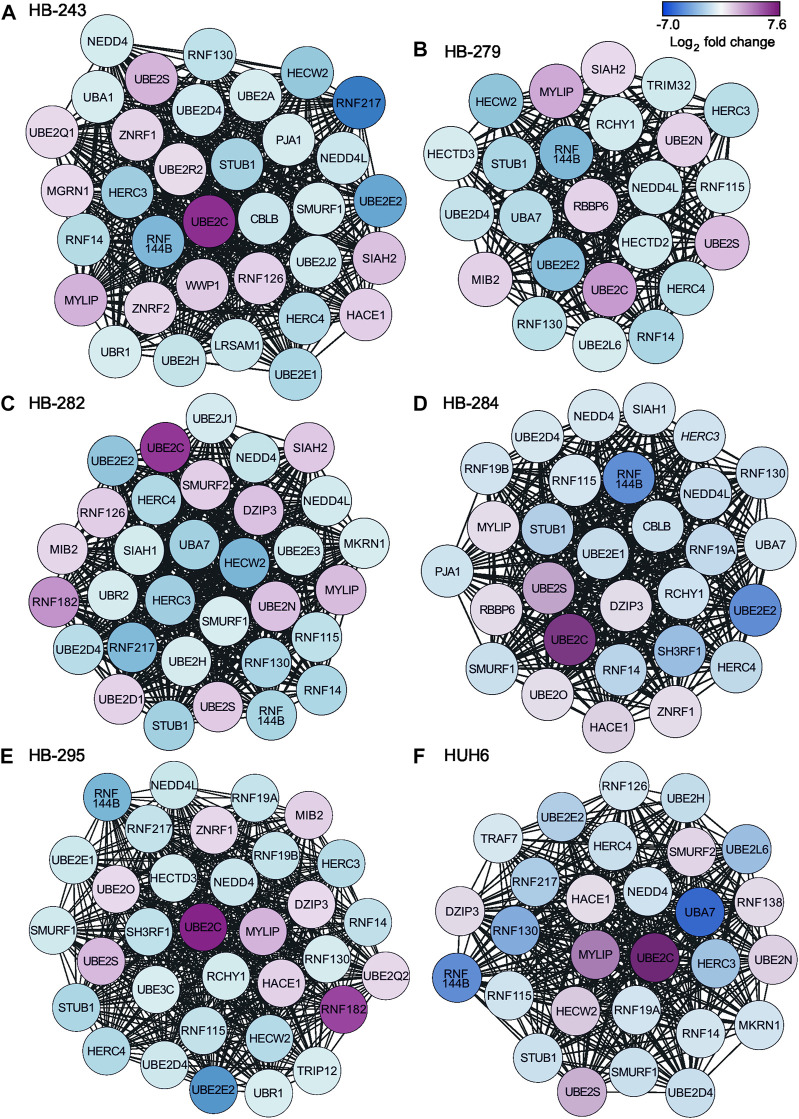
Highest-scoring clusters found in PPI networks of differentially expressed metabolic genes in each cell line. Clusters were ranked based on their score using MCODE plugin of Cytoscape. HB-243 **(A)**, HB-279 **(B)**, HB-282 **(C)**, HB-284 **(D)**, HB-295 **(E)**, and HUH6 **(F)**.

Protein-to-protein interaction (PPI) networks describe the physical contact of proteins within cells. PPI networks were constructed to better understand the changes in cell physiology represented by transcriptome analysis in HB. Lists of differentially expressed metabolic genes in each cell line (Supplementary Table S3) were used to construct the PPI networks using the STRING database. Highly interconnected areas (clusters) in each PPI-network were identified, scored, and ranked on size and density. The highest-scoring cluster of each cell line is shown in Figures 4A–F. The scores of the top clusters were 34 (HB-243), 24 (HB-279), 32 (HB-282), 29 (HB-284), 33 (HB-295) and 28 (HUH6). Upregulated genes found in one or more of these clusters included *UBE2C, UBE2D1, UBE2N, UBE2O, UBE2Q1, UBE2Q2, UBE2R2, UBE2S, DZIP3, HACE1, MGRN1, MIB2, MYLIP, RBBP6, RNF126, RNF138, RNF182, SIAH2, SMURF2, WWP1, ZNRF1,* and *ZNRF2.* Downregulated genes included *CBLB, HECTD2, HECTD3, HECW2, HERC3, HERC4, LRSAM1, MKRN1, NEDD4, NEDD4L, PJA1, RCHY1, RNF115, RNF130, RNF14, RNF144B, RNF19A, RNF19B, RNF217, SH3RF1, SIAH1, SMURF1, STUB1, TRAF7, TRIM32, TRIP12, UBA1, UBA7, UBE2A, UBE2D4, UBE2E1, UBE2E2, UBE2E3, UBE2H, UBE2J1, UBE2J2, UBE2L6 UBE3C, UBR1,* and *UBR2.*

### 3.4 KEGG and GO analyses of the highest-ranking clusters

**FIGURE 5:**
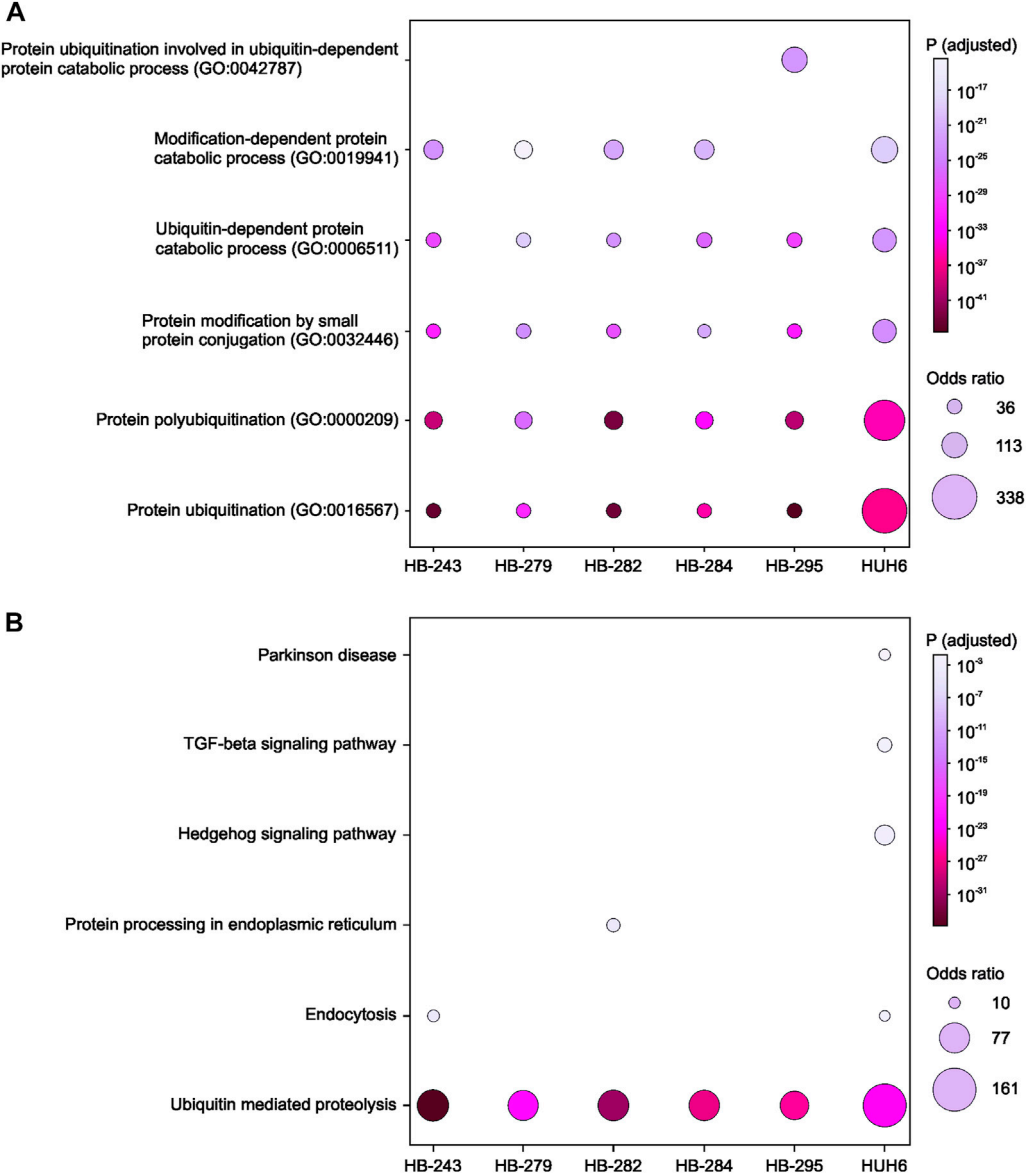
KEGG and GO analyses for enriched pathways. Genes in each cell line’s top-ranking PPI clusters were analyzed to find common enriched pathways. Statistically significant (*p* < 0.05) GO biological processes **(A)** and KEGG pathways **(B)** are shown for each cell line.

Next, to identify enriched pathways and gene sets, the list of protein-coding genes in each cell line’s highest-scoring PPI cluster were uploaded to the online tool Enrichr and the results ranked by *p*-value. The top 5 most statistically significant GO-terms in the highest-ranking clusters were protein ubiquitination, protein polyubiquitination, protein modification by small protein conjugation, modification-dependent protein catabolic processes, and protein ubiquitination involved in ubiquitin-dependent protein catabolic processes (Figure 5A). The corresponding KEGG-terms were ubiquitin mediated proteolysis, endocytosis, protein processing in endoplasmic reticulum, Hedgehog signaling pathway, TGF-β signaling pathway, and Parkinson’s disease (Figure 5B).

### 3.5 Validation of findings with HB patient microarray dataset

**FIGURE 6:**
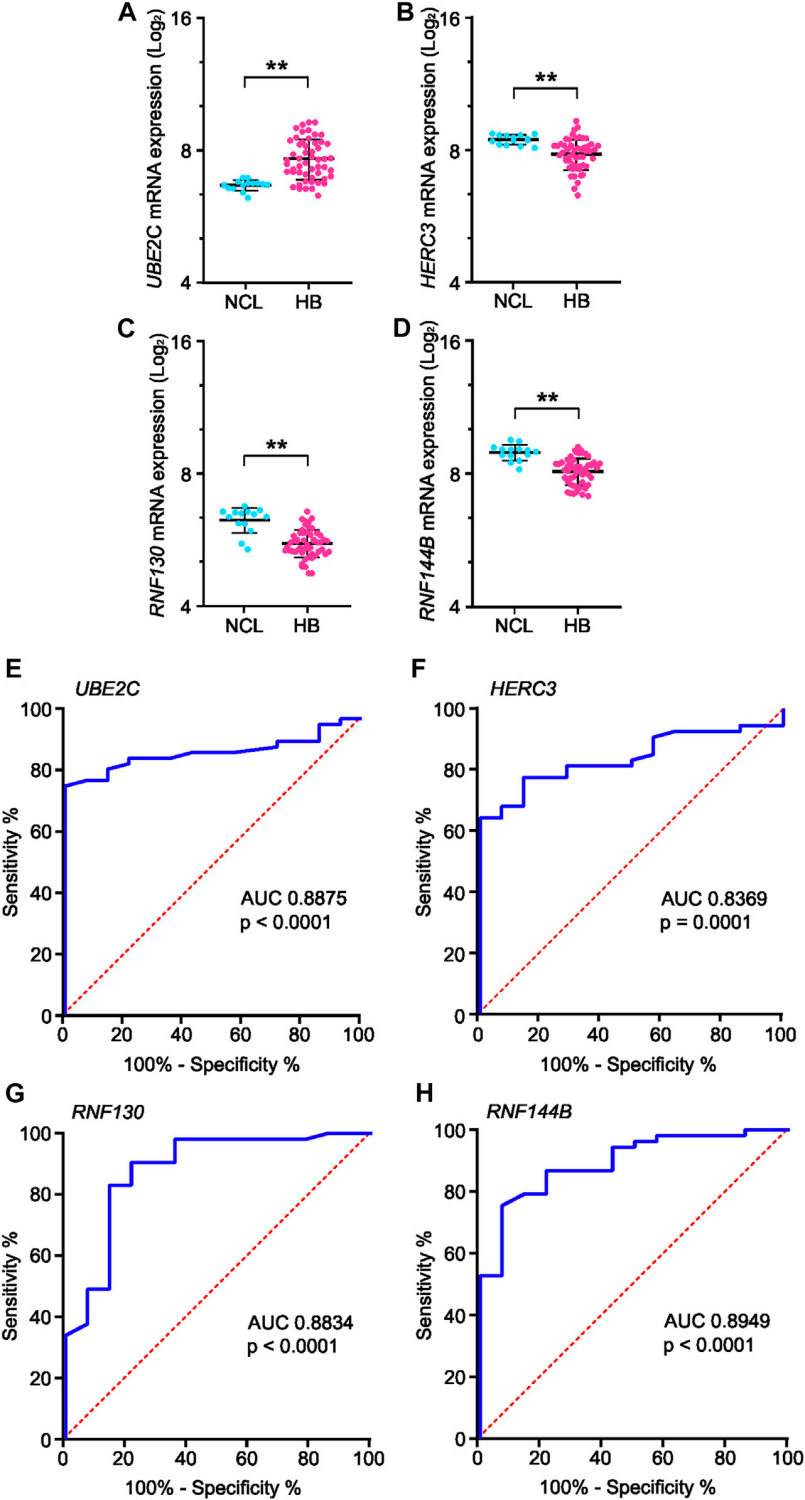
mRNA expression and ROC curve analysis of the four key genes in GSE131329 dataset. Expression of *UBE2C* **(A)**, *HERC3* **(B)**, *RNF130* **(C)** and *RNF144B* **(D)** in HB samples compared to noncancerous liver samples on mRNA level. ROC curve analysis of *UBE2C* **(E)**, *HERC3* **(F)**, *RNF130* **(G)** and *RNF144B* **(H)** assessing the suitability of each gene for discrimination of noncancerous liver (NCL) and HB. ** = *p* < 0.01. Grey dots represent gene expression of independent patients, the whiskers represent the first and third quartile, and the thick solid line is median **(A–D)**.

Genes present in all six of the highest-scoring clusters in the PPI-network were *RNF130, UBE2E2, UBE2C, RNF14, HERC3, HERC4, STUB1, UBE2S, RNF144B, MYLIP,* and *UBE2D4*. Of these, four genes—*RNF130, UBE2C, HERC3,* and *RNF144B*—were found to be significantly altered in the GSE131329 microarray dataset. This dataset includes 53 HB tissue samples and 14 noncancerous liver (NCL) tissue samples collected at the time of surgery from HB patients. *RNF144B*, *RNF130,* and *HERC3* mRNA expression was downregulated compared to the normal liver samples (log_2_FC −0.84, adj. *p*-value 1.10*10^−5^; log_2_FC −0.6, adj. *p*-value 3.0*10^−06^, and log_2_FC −0.62, adj. *p*-value 0.00186, respectively), whereas *UBE2C* mRNA expression was upregulated in HB samples (log_2_FC 1.0; adj. *p*-value 0.0001). RNF144B, RNF130, and HERC3 act as a ubiquitin ligases and UBE2C is an ubiquitin conjugating enzyme. Expression of these 4 genes in both HB and NCL groups is shown in Figures 6A–D.

### 3.6 Clinical analyses of potential key genes *UBE2C, RNF130, HERC3,* and *RNF144B*

To analyze the suitability of each selected gene (*UBE2C*, *RNF130*, *HERC3*, and *RNF144B*) to discriminate normal control liver samples (NCL) and HB, we performed ROC curve analysis. The area under curve (AUC) was 0.8875 for *UBE2C* (Figure 6E, 95% CI 0.81–0.96), 0.8369 for *HERC3* (Figure 6F, 95% CI 0.74–0.93), 0.8834 for *RNF130* (Figure 6G, 95% CI 0.77–0.99), and 0.8949 for *RNF144B* (Figure 6H, 95% CI 0.81–0.98). Next, we assessed the association of each of these genes with distant metastasis status, occurrence of events, and overall survival. High *UBE2C* mRNA expression was linked with distant metastasis (*p*-value <0.01), events (*p*-value <0.05), and death (*p*-value <0.01) (Table 2). Downregulation of *HERC3* and *RNF144B* was associated with occurrence of events (*p*-values <0.05) (Table 2). *RNF130* expression did not show a statistically significant association with any of the studied variables (Table 2). Clinical information adapted from GSE131329 dataset is summarized in Table 3.

**TABLE 2:** Association of UBE2C, HERC3, RNF130, and RNF144B mRNA expression (log2) with clinical course of the disease assessed with Mann-Whitney U test.

	*UBE2C*	*HERC3*	*RNF130*	*RNF144B*
Distant metastasis NO (*n = 39*) Median mRNA exp	7.330	7.940	9.400	8.240
Distant metastasis YES (*n = 14*) Median mRNA exp	8.295	7.440	9.470	7.765
*p*-value	0.0046	0.0682	0.5592	0.0874
Event-free YES (*n = 32*) Median mRNA exp	7.325	8.060	9.460	8.310
Event-free NO (*n = 21*) Median mRNA exp	7.930	7.580	9.330	7.860
*p*-value	0.0303	0.0247	0.6619	0.0130
Overall survival ALIVE (*n = 38*) Median mRNA exp	7.325	8.005	9.425	8.280
Overall survival DEAD (*n = 15*) Median mRNA exp	8.240	7.560	9.510	7.840
*p*-value	0.0095	0.0581	0.4966	0.0269

**TABLE 3:** Clinical information of GSE131329 dataset. (HB = hepatoblastoma; NCL = non-cancerous liver).

	HB (*n* = 53)	NCL (*n* = 17)
Median age, months	22 (0–109)	17 (5–98)
Sex FEMALE, n (%)	47.2	57.1
Sex MALE, n (%)	52.8	42.9
PRETEXT = 1, n	9	—
PRETEXT = 2, n	15	—
PRETEXT = 3, n	18	—
PRETEXT = 4, n	11	—
Distant metastasis NO, n	39	—
Distant metastasis YES, n	14	—
Event-free NO, n	21	—
Event-free YES, n	32	—
Overall survival, alive (%)	71.7	—

### 3.7 UBE2C associated protein interactions and gene co-expression analysis

**FIGURE 7:**
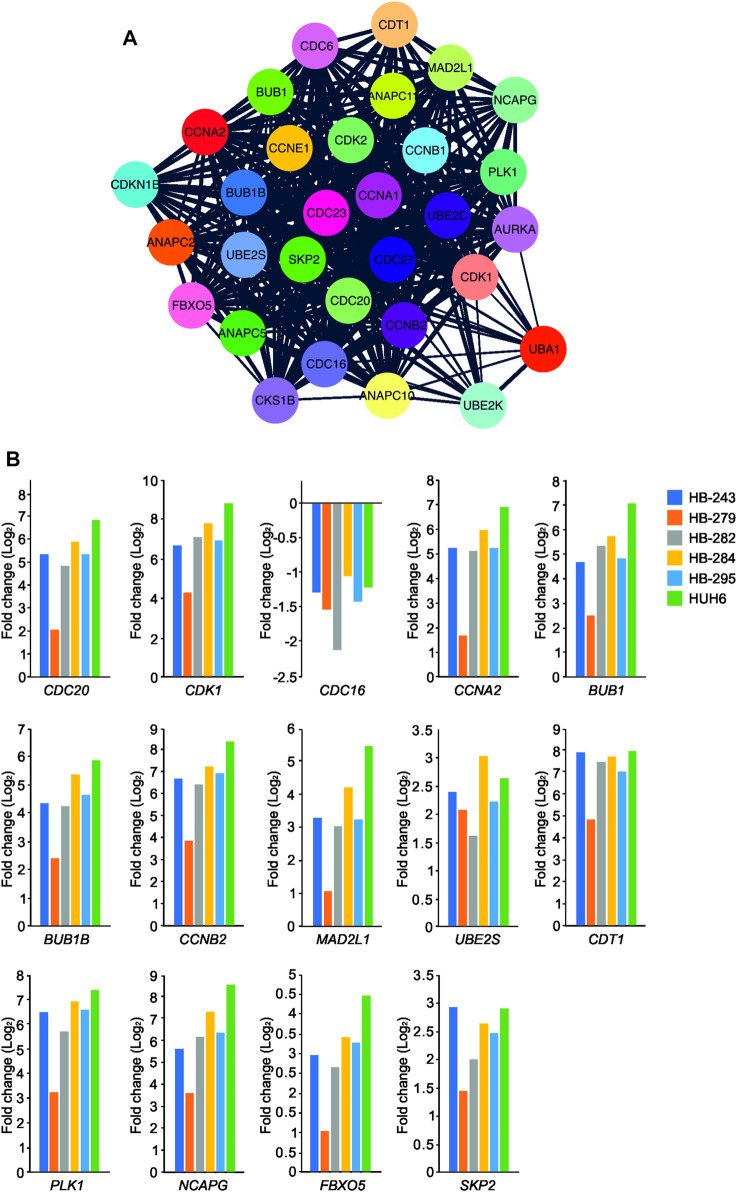
UBE2C protein-protein interaction network. UBE2C associated protein-protein interactions **(A)**. The fold change of UBE2C interacting DEGs (in relation to primary hepatocyte) emerging from our RNA sequencing analysis **(B)**.

To analyze the functional enrichment of UBE2C specific protein interactions in general, STRING network analysis was carried out. UBE2C was used as the input protein. Thirty proteins were interacting with UBE2C when the cut-off was set to 0.4 (Figure 7A). Next, we assessed the level of differential RNA expression of these UBE2C interacting proteins in our RNA sequencing data of the six HB cell models. Of these 30 proteins, fourteen were found to be significantly differentially expressed on RNA level in all 6 HB models (Figure 7B).

Gene co-expression analysis was conducted for *UBE2C* using the online tool GeneFriends to further explore the relationship between *UBE2C* and related genes. Using *UBE2C* as the seed gene, top 10 co-expressed genes with the highest Pearson correlation values were *CCNB2, TOP2A, NUSAP1, CKS2, NUF2, CDK1, NEK2, PTTG1, CKS1B,* and lncRNA *RP11-102C16.3* (Supplementary Figure S1A–B). When comparing these protein interactions and gene co-expression results of UBE2C, three genes/proteins, CKS1B, CCNB2 and CDK1, were present in both networks.

### 3.8 Immunohistochemical staining of UBE2C in HB patient samples

**FIGURE 8:**
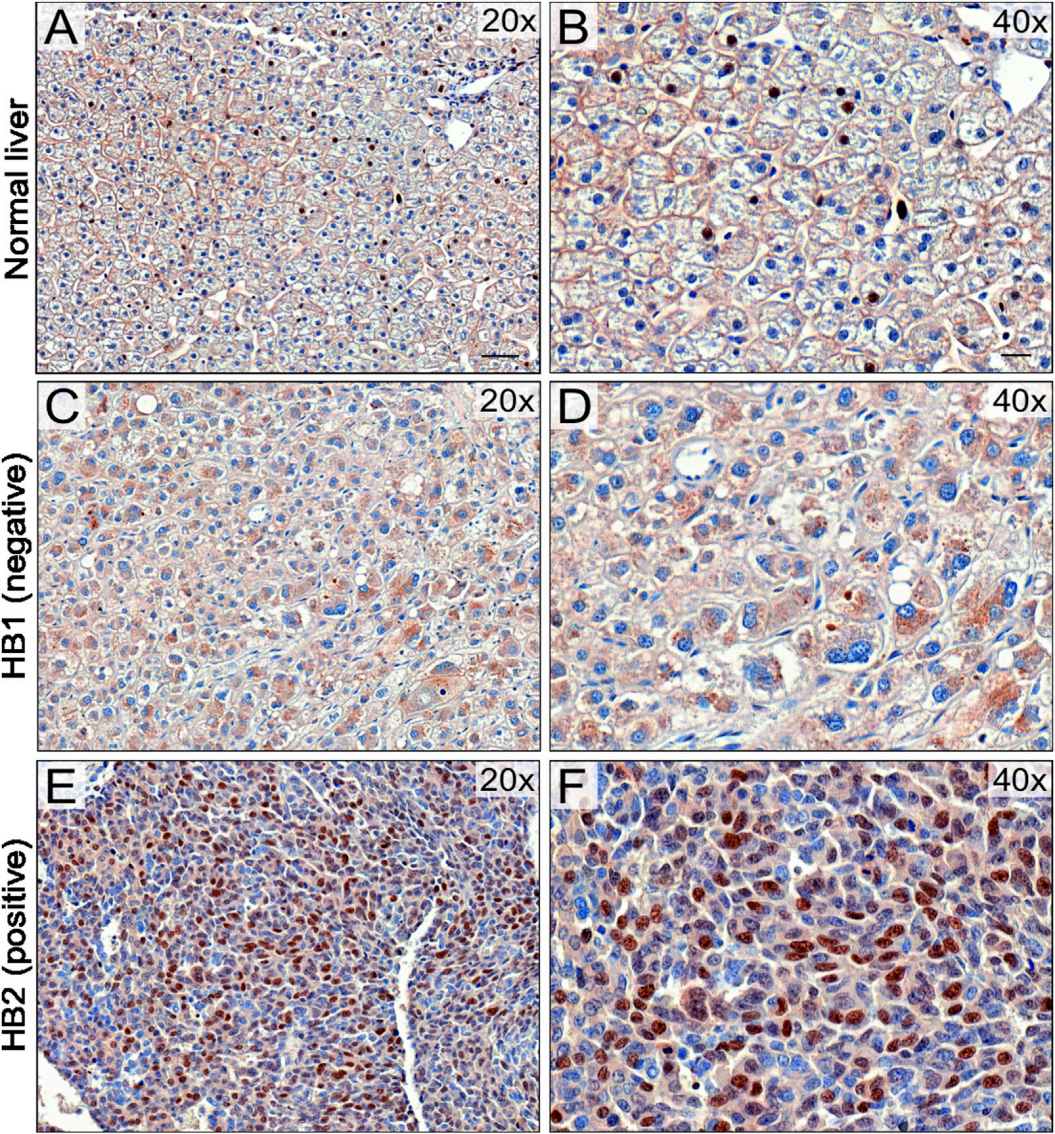
UBE2C protein expression in HB tissues. Immunohistochemical staining of UBE2C in 6 normal liver (NL) samples and 25 hepatoblastoma (HB) samples was done. Representative image of normal liver stained with UBE2C with ×20 **(A)** and ×40 **(B)** magnification. Representative image of HB liver staining negative for UBE2C with ×20 **(C)** and ×40 **(D)** magnification. Representative sample of HB liver staining positive for UBE2C ×20 **(E)** and ×40 **(F)** magnification. Scale bars = 50 µm **(B,D)** and 20 µm **(C,E)**.

To validate UBE2C protein expression in HB patient samples, immunohistochemical staining was performed on 6 NL (Table 4) and 25 HB (Table 5) samples. One of the 6 NL samples showed positive *UBE2C* staining on the cell membranes (Figures 8A, B). Of the HB samples, 5 were considered UBE2C-negatives (Figures 8C, D) and 20 UBE2C positive (Figures 8E, F). Compared to NL samples, staining for UBE2C in HB cells appeared stronger and localized to nuclei rather than the cell membrane.

**TABLE 4:** Liver samples from donors used in IHC.

Sample	UBE2C staining	Age at death (years)	Sex	Cause of death
NL1	−	3	M	anoxia, heart-related
NL2	−	8	M	traumatic brain injury
NL3	+	11	F	anoxia, trauma-related
NL4	−	26	F	traumatic brain injury
NL5	−	14	M	traumatic brain injury
NL6	−	16	F	spontaneous subdural hemorrhage

**TABLE 5:** Clinical information of HB patient tissue samples used in IHC.

Sample	UBE2C staining	Age at tx/res (years, age group)	Risk	Histology	Sex
HB1	+	3–7	high	NA	F
HB2	+	>7	high	epithelial, macrotrabecular	M
HB3	+	1–3	high	fetal epithelial, well differentiated	F
HB4	+	1–3	high	embryonal and fetal epithelial	F
HB5	+	>7	high	fetal epithelial	M
HB6	+	1–3	standard	mixed epithelial and mesenchymal	M
HB7	+	1–3	standard	fetal epithelial	M
HB8	−	3–7	high	fetal epithelial	F
HB9	+	3–7	high	epithelial	F
HB10	+	1–3	standard	fetal epithelial	M
HB11	+	>7	high	fetal epithelial	F
HB12	+	>7	high	fetal epithelial	M
HB13	+	<1	standard	fetal, teratoid features	M
HB14	+	1–3	standard	fetal epithelial	M
HB15	−	1–3	high	mixed epithelial and mesenchymal with teratoid features	F
HB16	+	<1	high	mixed epithelial and mesenchymal	M
HB17	+	1–3	high	embryonal and fetal epithelial	M
HB18	+	>7	high	embryonal and fetal epithelial	F
HB19	+	3–7	high	fetal epithelial	M
HB20	-	>7	high	embryonal and fetal epithelial	F
HB21	+	3–7	high	embryonal and fetal epithelial	F
HB22	−	3–7	high	fetal epithelial	M
HB23	−	3–7	high	embryonal epithelial	F
HB24	+	3–7	high	mixed	M
HB25	+	3–7	high	fetal epithelial, well differentiated	M

### 3.9 *UBE2C* silencing decreases cell viability and migration in HUH6 and HB-243 HB cell models

**FIGURE 9:**
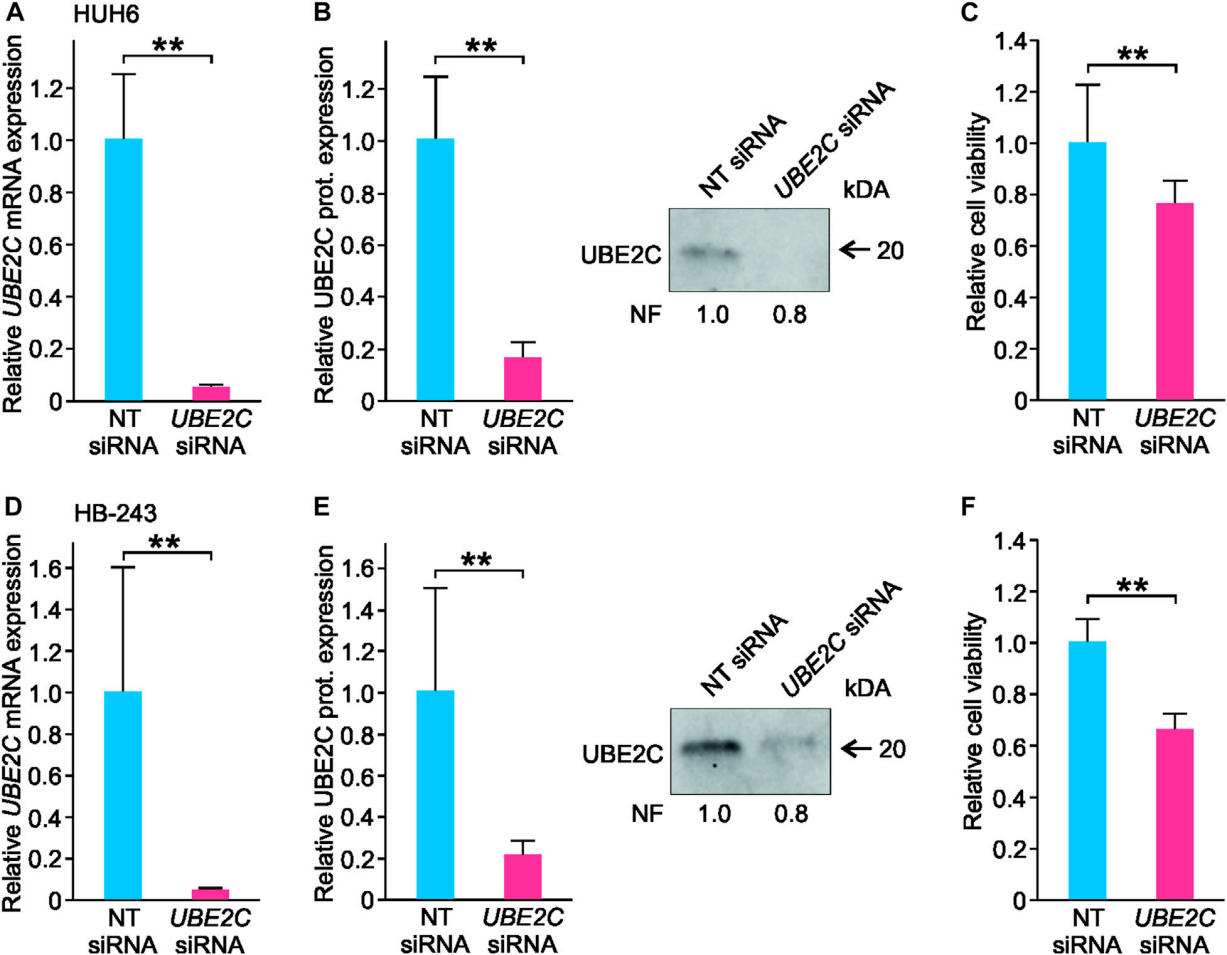
Knockdown of *UBE2C* and its effect on HUH6 and HB-243 cell viability. Following the siRNA transfection, *UBE2C* mRNA expression in HUH6 was reduced by 95% **(A)** and protein expression by 80% **(B)** compared to cells transfected with non-targeting (NT) siRNA. In HB-243, *UBE2C* mRNA expression was reduced by 98% **(D)** and protein expression by 80% **(E)**. WST-1 assay showed a 24% decrease in HUH6 **(C)** and 44% in HB-243 **(F)** in cell viability after *UBE2C* silencing. Bar plots are presented as relative values of mean of three independent experiments ± RSD. **p-value <0.01, NT = non-targeting. Normalization factor (NF) describing the amount of total protein in lane in relation to other lanes is given beneath the bands **(B, E)**.

To explore UBE2C function *in vitro*, *UBE2C* was silenced in HUH6 and HB-243 cell lines using siRNA transfection. Non-targeting (NT) siRNAs were used as a control. After siRNA transfections, *UBE2C* expression was reduced 95% at mRNA and 80% at protein level in HUH6 (Figures 9A, B) and 98% at mRNA and 80% at protein level in HB-243. (Figures 9D, E). The effect of *UBE2C* knockdown on cell viability was evaluated using WST-1 assay. Relative cell viability decreased 24% in HUH6 (Figure 9C) and 44% in HB-243 (Figure 9F).

**FIGURE 10:**
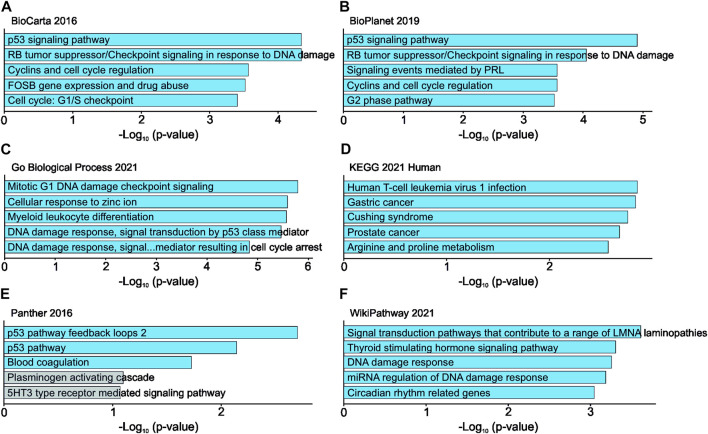
Effects of *UBE2C* knockdown in HUH6 cells on RNA level. *UBE2C* knockdown is linked with alterations in RNA expression of genes connected to cell cycle regulation and p53 signaling pathway. Top 5 pathways and ontologies ranked by *p*-value **(A–F)**.

RNA sequencing of *UBE2C* silenced HUH6 cells showed 111 differentially expressed genes (Supplementary Table S5). Top 5 pathways and ontologies matching these genes included cell cycle related processes such as p53 regulation, DNA damage response, and G1/S checkpoint (Figures 10A–F).

Effects of *UBE2C* knockdown in HUH6 cells were further evaluated using a clonogenic assay, which showed a statistically significant fall in cell number in *UBE2C* silenced cells with the well area covered by cells decreasing 35% (Supplementary Figure S2A–C). The effect of *UBE2C* silencing on HUH6 cell migration was assessed with transwell assay, which demonstrated a statistically significant 65% decrease in the number of migrated cells compared to NT siRNA treated cells (Supplementary Figure S2D–F).

## 4 Discussion

Metabolic derangements have been associated with enhanced tumorigenesis and cancer progression in several tumor entities (Faubert et al., 2020), and these tumor-specific changes can be exploited to develop targeted therapies (Sullivan et al., 2016). In our RNA sequencing analysis of HB cell models, ubiquitination emerged as the most significantly altered metabolic pathway. The expression of three ubiquitin ligases (*HERC3*, *RNF130*, and *RNF144A*) and one ubiquitin conjugating enzyme (*UBE2C*) was significantly dysregulated in all studied HB models. These four genes were assessed more thoroughly in a HB patient dataset to validate their significance in the clinical setting. We noticed a remarkable association between high *UBE2C* expression and aggressive disease in the particular HB patient cohort.

Ubiquitination is a crucial mechanism for the degradation of short-lived proteins like those involved in cell cycle regulation (Guo et al., 2019; Zhang et al., 2021). In addition to protein degradation, ubiquitination regulates DNA repair, translation, and inflammation (Miranda and Sorkin, 2007). The three main steps in ubiquitination are activation (performed by ubiquitin-activating enzymes, E1s), conjugation (ubiquitin-conjugating enzymes, E2s), and ligation (ubiquitin-ligating enzymes, E3s) (Komander and Rape, 2012). Ubiquitination and deubiquitination are known to be modulated during cancer progression (Sun et al., 2020), and high *UBE2C* expression portends poor survival in various cancers including node-positive breast cancer (Loussouarn et al., 2009) and ovarian carcinomas (Berlingieri et al., 2007). The magnitude of *UBE2C* mRNA overexpression in HB cell lines was striking (up to 128-fold higher than primary hepatocytes), and *UBE2C* expression in HB clinical specimens was associated with increased risk of distant metastasis, events, and death. Our findings echo a recent study showing that *UBE2C* expression may be used as a diagnostic biomarker in hepatocellular carcinoma, the most frequent liver cancer in adults (Gao et al., 2021).

In HB tissue samples, we observed predominantly nuclear localization of UBE2C protein. In other cancers, both nuclear and cytoplasmic UBE2C immunoreactivity have been observed, but the functional significance of this is unclear (Shen et al., 2013; Ma et al., 2016; Palumbo et al., 2016). Kraft *et al.* showed that strong nuclear expression of UBE2C was linked with higher mitosis rate in melanoma suggesting that UBE2C localization in nuclei may be at least partially related to its role in the regulation of cell cycle associated proteins (Kraft et al., 2017).

It has previously been shown that in cancer cells UBE2C plays an important role in facilitation of protein degradation and dysregulation of the cell cycle (Sun et al., 2020). UBE2C overexpressing cells have the ability to override mitotic spindle checkpoints, which may lead to loss of genomic stability, a characteristic of cancer (Reddy et al., 2007). UBE2C is also suggested to be a potential oncogene enhancing migration and invasion in hepatocellular carcinoma (Xiong et al., 2019). Consistently, we demonstrated that knockdown of *UBE2C* resulted in a decrease in HB viability, and preliminary results suggest that it could also have a negative effect on HB cell migration. Our RNA sequencing results supported the hypothesis that UBE2C participates in cell cycle regulation in HB. After *UBE2C* knockdown, we observed alterations in mRNA expression of *CDKN1A*, *CDK, PIK3C2B, PIDD1,* and *E2F2* genes which are known to participate in cell cycle regulation and the p53 signaling pathway. Changes in mRNA expression, however, were rather subtle. Effects of *UBE2C* knockdown on cell cycle were, however, not assessed with *in vitro* experiments in this article. Given the role of UBE2C as a post-translational factor rather than a direct regulator of gene expression, proteomics analysis could be conducted in future experiments. UBE2C overexpression has also previously been linked to increased ubiquitination and subsequent degradation of the tumor suppressor p53 in endometrial cancer (Liu et al., 2020). A novel therapy aimed at enhancing p53 activity has been suggested to be a potential treatment alternative for HB (Woodfield et al., 2021). If UBE2C participates in post-translational regulation of p53 expression in HB, its inhibition could lead to reactivation of p53.

Aurora Kinase A (AURKA), a serine/threonine kinase, has a critical role in regulating cell cycle and mitosis (Du et al., 2021). Expression of *AURKA* has been shown to be significantly higher in HB than in normal liver (Tian et al., 2021). In our study, *AURKA* was significantly upregulated in all studied HB cell lines. Treatment modalities targeting AURKA, such as alisertib, have shown promising results in preclinical studies of HB (Tan et al., 2020). Interestingly, increased AURKA expression has been demonstrated to correlate with upregulated *UBE2C* in cancer cells (http://gepia.cancer-pku.cn) (Naso et al., 2021). Furthermore, inhibition of *UBE2C* expression was shown to reduce the level of phosphorylation of AURKA and impair cell viability in gastric adenocarcinoma cells (Wang et al., 2017).

UBE2C links to another key HB gene, cyclin-dependent kinase 1 (*CDK1*) (Aghajanzadeh et al., 2020; Sun et al., 2021; Tian et al., 2021). CDK1 functions as a serine/threonine kinase and, like AURKA, plays an important role in cell cycle regulation. CDK1 has been reported to be upregulated in various cancers including hepatocellular carcinoma (Zhou et al., 2019). Consistent with previous studies, our RNA-sequencing results showed that *CDK1* was highly upregulated in HB cell models. *CDK1* siRNA knockdown was shown to inhibit the growth and invasiveness of HUH6 HB cells (Tian et al., 2021). A study of ovarian cancer cells showed that high UBE2C expression correlated with expression of CDK1. Knockdown of *UBE2C* induced G2/M arrest in the cells, which led to decreased CDK1 expression (Li et al., 2020). In our study, knockdown of *UBE2C* in HUH6 cells increased cyclin-dependent kinase inhibitor 1 (*CDKN1A*) expression and decreased cyclin-dependent kinase 2 (*CDK2*) expression at mRNA level, both of these having a role in G1/S transition. *CDK1* expression was not significantly altered.

There is some evidence that *UBE2C* overexpression impacts chemoresistance. Downregulation of *UBE2C* reversed resistance to cisplatin in ovarian cancer cell models (Li et al., 2020). UBE2C inhibition has been shown to increase doxorubicin sensitivity in breast cancer cells *in vitro* (Rawat et al., 2013). Cisplatin and doxorubicin are both widely used in HB management. In addition to targeted treatment, UBE2C expression status could thus be utilized in the evaluation of treatment resistance to conventional chemotherapy in HB. The proteasome inhibitor bortezomib slows HB progression *in vitro* and *in vivo* (Hooks et al., 2018). In colorectal carcinoma, bortezomib treatment has been demonstrated to downregulate UBE2C expression leading to decreased cell viability via stabilizing mitotic cyclins and inhibiting cell cycle progression (Bavi et al., 2011). Thus, high UBE2C expression could identify HB patients who may benefit from bortezomib treatment.

There are some limitations to this study. The reader should note that in this article, both mRNA- and protein expression of the genes in question are being used. While changes in mRNA-expression often correlate with changes in protein expression, this is not always the case given the several factors affecting the translation process and the final amount of protein in the tissue. This should be kept in mind while interpretating the results. In this study we have used immunohistochemistry and Western blotting to determine the protein expression levels of UBE2C in HB tissues. More extensive proteomics would, however, be required to further elucidate the actual protein expression levels of all the genes related to *UBE2C*. Liver matures throughout childhood, and the use of primary hepatocytes from adult donor as control cells in RNA sequencing analyses of the PDX models may have impacted our results. The noted effects of *UBE2C* silencing on mRNA expression level of cell cycle regulating genes should be further validated with *in vitro* and *in vivo* experiments in order to properly assess the effects on cell cycle. The number of HB patient samples available for this study limited clinical analyses and conclusions. This is unfortunately the case with most studies concerning HB, since the prevalence of HB is low and the availability of samples therefore limited.

Given the promising role of ubiquitin system as a target of new cancer treatments, the role and function of UBE2C in HB progression should be investigated further. The possible role of UBE2C and other ubiquitination-mediating enzymes in drug resistance is also intriguing. One possible technique that could be utilized is single-cell RNA sequencing (scRNAseq). Previously, Bondoc et al. have characterized HB tumor cell populations and identified driver tumor cell clusters using scRNAseq (Bondoc et al., 2021). Given the advantages of the technique, analysis of scRNAseq could provide new insight.

Taken together, we found that metabolic alterations taking place in HB tumors are diverse and that ubiquitination-related factors may have a significant role in HB progression. Notably, UBE2C expression was highly upregulated in all six HB cell lines as well as in patient samples at both mRNA and protein level. *In vitro* knockdown of *UBE2C* resulted in decreased cell division and motility. Moreover, high UBE2C expression was associated with inferior patient survival. These findings may be brought to the clinic to identify the high-risk HB patients for earlier treatment interventions.

## Figures and Tables

**Table:** 

**AURKA**	aurora kinase A
**CDK1**	cyclin-dependent kinase 1
**CDKN1A**	cyclin-dependent kinase inhibitor 1A
**CYP2C18**	cytochrome P450 Family 2 subfamily C member 18
**DEG**	differentially expressed gene
**DPEP1**	dipeptidase 1
**E2F2**	E2F transcription factor 2
**FFPE**	formalin-fixed paraffin-embedded
**GLUT3**	glucose transporter 3
**GO**	gene ontology
**HB**	hepatoblastoma
**HERC3**	HECT and RLD domain containing E3 ubiquitin protein ligase 3
**IGDCC3**	immunoglobulin superfamily DCC subclass member 3
**KEGG**	kyoto encyclopedia of genes and genomes
**NCL**	non-cancerous liver
**NL**	normal liver
**NNMT**	nicotinamide N-methyltransferase
**NT**	non-targeting
**ODAM**	odontogenic ameloblast-associated protein
**PBS**	phosphate-buffered saline
**PCYT1B**	phosphate cytidylyltransferase 1B
**PIDD1**	P53-onduced death domain protein 1
**PDX**	patient-derived xenografts
**PIK3C2B**	phosphatidylinositol-4-phosphate 3-kinase catalytic subunit type 2 beta
**PPI**	protein-protein interaction
**p53**	tumor protein p53
**RBP2**	retinol binding protein 2
**RNF130**	ring finger protein 130
**RNF144B**	ring finger protein 144B
**ROC**	receiver operating characteristic
**SAA1**	serum amyloid A1
**SAA2**	serum amyloid A2
**siRNA**	small interfering RNA
**SLC10A1**	solute carrier family 10 member 1
**TRIM71**	tripartite motif containing 71
**UBE2C**	ubiquitin conjugating enzyme E2 C
**VMH**	virtual metabolic human
**VNN1**	vanin1

## Data Availability

The datasets presented in this study can be found in online repositories. The names of the repository/repositories and accession number (s) can be found at https://www.ncbi.nlm.nih.gov/geo/ (GSE140520, GSE83518, GSE131329) and https://ega-archive.org/ (EGAS00001004827/EGAD00001006621).
