# Supplementary material for: UBE2C expression is elevated in hepatoblastoma and correlates with inferior patient survival
Source: Front Genet. 2023 Jun 12;14:1170940. doi: 10.3389/fgene.2023.1170940 (PMC10291054; doi:10.3389/fgene.2023.1170940)
Supplement: Supplementary file 1 [file DataSheet2.PDF]

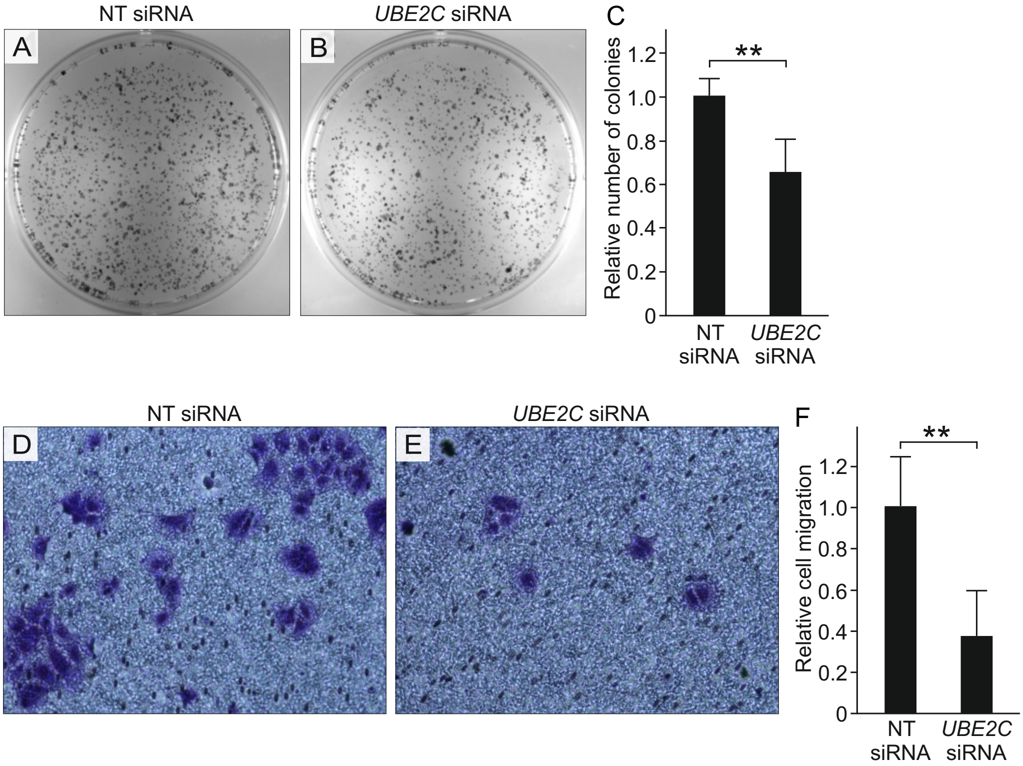

Supplementary Figure 2. *UBE2C* knockdown leads to decreased colony formation and migration in HUH6 cells. The number of colonies was decreased approximately 35% after *UBE2C* knockdown (A-C). A decreased number of *UBE2C* siRNA treated cells migrated through the transwell membrane when compared to control cells (D-F). The relative cell migration was 65% lower in *UBE2C* silenced cells compared to NT treated cells. Bar plots are presented as relative values of mean of three independent experiments ± RSD. **p-value < 0.01, NT = non-targeting.
