# Supplementary material for: UBE2C expression is elevated in hepatoblastoma and correlates with inferior patient survival
Source: Front Genet. 2023 Jun 12;14:1170940. doi: 10.3389/fgene.2023.1170940 (PMC10291054; doi:10.3389/fgene.2023.1170940)
Supplement: Supplementary file 4 [file DataSheet1.PDF]

A

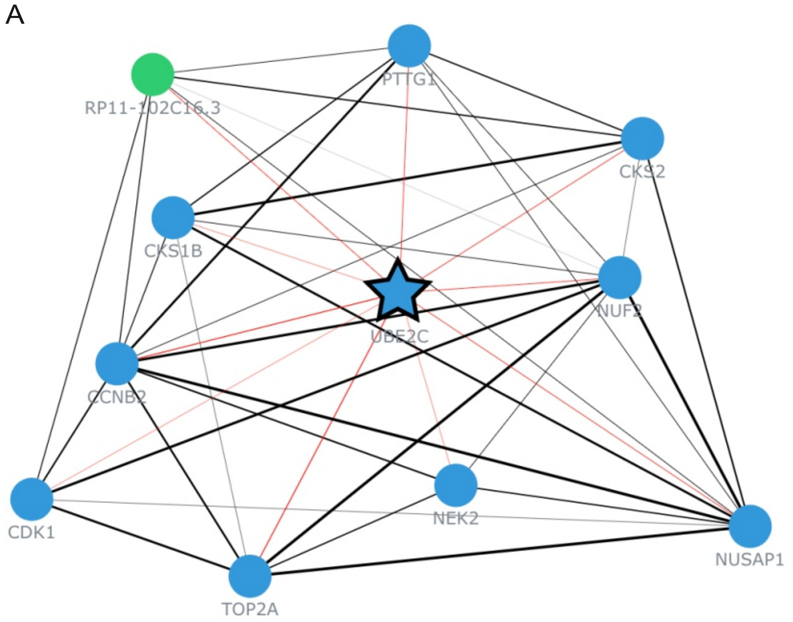

B

Ensembl ID	Gene Symbol	Pearson Correlation	Gene Biotype	Chromosome	Annotation
ENSG00000131747	TOP2A	0.778646975278983	protein_coding	17	StAR related lipid transfer domain containing 3
ENSG00000157456	CCNB2	0.775034220451122	protein_coding	15	family with sequence similarity 81 member A
ENSG00000137804	NUSAP1	0.771144016513867	protein_coding	15	NADH:ubiquinone oxidoreductase complex assembly factor 1
ENSG00000227907	RP11-102C16.3	0.768333743638495	lncRNA	1	uncharacterized LOC441072
ENSG00000123975	CKS2	0.767875647323378	protein_coding	9	dynein assembly factor with WD repeats 1
ENSG00000164611	PTTG1	0.766437494592757	protein_coding	5	calcium modulating ligand
ENSG00000143228	NUF2	0.763651755122356	protein_coding	1	regulator of G protein signaling 5
ENSG00000173207	CKS1B	0.758095498652185	protein_coding	1	ATP binding cassette subfamily D member 2
ENSG00000170312	CDK1	0.755179358322755	protein_coding	10	ubiquitin B
ENSG00000117650	NEK2	0.755095011799378	protein_coding	1	ribosomal protein S6 kinase A1

Supplementary Figure 1. Gene co-expression analysis for UBE2C was conducted using GeneFriends online tool. Top 10 co-expressed genes ranked by Pearson correlation are shown (A). In the co-expression network, seed gene *UBE2C* is marked with a star. Blue nods represent protein-coding genes while the green node is a long non-coding RNA (lncRNA) (A). Pearson correlation values for each of the top 10 co-expressed genes and *UBE2C* are shown in table (B).
